# COVID-19 and the cardiovascular system: a study of pathophysiology and interpopulation variability

**DOI:** 10.3389/fmicb.2023.1213111

**Published:** 2023-06-07

**Authors:** Yifan Zhao, Xiaorong Han, Cheng Li, Yucheng Liu, Jiayu Cheng, Binay Kumar Adhikari, Yonggang Wang

**Affiliations:** ^1^Department of Cardiovascular Center, The First Hospital of Jilin University, Changchun, China; ^2^Department of Special Care Center, Fuwai Hospital, National Clinical Research Center for Cardiovascular Diseases, National Center for Cardiovascular Diseases, Chinese Academy of Medical Science and Peking Union Medical College, Beijing, China; ^3^Department of Family and Community Medicine, Feinberg School of Medicine, McGaw Medical Center of Northwestern University, Chicago, IL, United States; ^4^Department of Cardiology, Nepal Armed Police Force Hospital, Kathmandu, Nepal

**Keywords:** SARS-CoV-2, COVID-19, cardiovascular disease, metabolic syndrome, endothelium, ACE2

## Abstract

The severe acute respiratory syndrome coronavirus 2 (SARS-CoV-2) infection in humans can lead to various degrees of tissue and organ damage, of which cardiovascular system diseases are one of the main manifestations, such as myocarditis, myocardial infarction, and arrhythmia, which threaten the infected population worldwide. These diseases threaten the cardiovascular health of infected populations worldwide. Although the prevalence of coronavirus disease 2019 (COVID-19) has slightly improved with virus mutation and population vaccination, chronic infection, post-infection sequelae, and post-infection severe disease patients still exist, and it is still relevant to study the mechanisms linking COVID-19 to cardiovascular disease (CVD). This article introduces the pathophysiological mechanism of COVID-19-mediated cardiovascular disease and analyzes the mechanism and recent progress of the interaction between SARS-CoV-2 and the cardiovascular system from the roles of angiotensin-converting enzyme 2 (ACE2), cellular and molecular mechanisms, endothelial dysfunction, insulin resistance, iron homeostasis imbalance, and psychosocial factors, respectively. We also discussed the differences and mechanisms involved in cardiovascular system diseases combined with neocoronavirus infection in different populations and provided a theoretical basis for better disease prevention and management.

## 1. Introduction

The global pandemic of the new coronavirus poses a great challenge to health care systems. In addition to the direct physical damage, the economic and social stress caused by the rampant virus is affecting the psychological health of people around the world to varying degrees (Liu D. et al., 2021; Isath et al., 2023a). Different cardiovascular system (CVD) complications can occur after the severe acute respiratory syndrome coronavirus 2 (SARS-CoV-2) infection, including myocarditis (0.128%–0.15%; Fairweather et al., 2023; Keller et al., 2023), stress cardiomyopathy (0.1%–5.6%; Chung et al., 2021; Davis et al., 2023), pulmonary embolism (PE; 1.9%; Hobohm et al., 2023), heart failure (3%–33%; Chung et al., 2021), and myocardial infarction (0.9%–11%; Chung et al., 2021). In this general situation, SARS-CoV-2 combined with cardiac disease can lead to a worse clinical prognosis, and patients usually have a higher mortality rate. For example, patients with combined myocarditis have a higher mortality rate than those without myocarditis (24.3% vs. 18.9%; Keller et al., 2023), and patients with combined pulmonary embolism have a significantly higher mortality rate (28.7%) than patients with neocoronavirus without combined PE (17.7%; Hobohm et al., 2023). Currently, a large number of studies have taken the first step toward revealing the interaction between the coronavirus disease 2019 (COVID-19) and the cardiovascular system, but some of the specific mechanisms are still unclear. Also, due to individualized differences, different populations tolerate and respond differently to the disease (Liu F. et al., 2021; Tobler et al., 2022). In this review, we seek to explore how cardiac structure and function are affected in the context of COVID-19 and to discuss inter-population differences in COVID-19 comorbid cardiovascular disease for better risk stratification and precise management in the post-epidemic period.

## 2. Mechanism of interaction between SARS-CoV-2 and the cardiovascular system

### 2.1. ACE2

ACE2 is an important receptor for neocoronavirus invasion of human cells and is widely distributed in various organs of the body, such as the lung, heart, gastrointestinal tract, and kidney, and it has a high affinity for the viral protein receptor-binding domain (RBD). The binding of the two induces spatial folding and conformational changes in the RBD, promotes membrane fusion, and induces viral entry into cells through endocytosis (Chung et al., 2021). ACE2 is an extremely important and beneficial biomolecule for the cardiovascular system, which can promote the conversion of AngII to Ang1-7, reduce cardiac pathological remodeling, and exert cardiovascular protective effects (Kuriakose et al., 2021). When ACE2 binds and interacts with SARS-CoV-2 spike (S) protein, the structural domain of metalloprotease protein-17 (ADAM17) is activated and cleaves the extracellular region of ACE2, leading to an increase in soluble ACE2 (sACE2) in the plasma and a decrease in cell surface ACE2 expression. In turn, a decrease in ACE2 not only amplifies the deleterious effects of AngII but also further upregulates ADAM17 expression, creating a vicious cycle (Lambert et al., 2005; Zipeto et al., 2020; Liu F. et al., 2021; García-Escobar et al., 2022).

There is a correlation between the tropism and severity of viral infections and the expression of host cell receptors. Increased expression of plasma soluble ACE2 was found in patients with myocardial infarction, atrial fibrillation, valvular disease, and heart failure, reflecting a higher basal ACE2 expression and increased susceptibility in this population (García-Escobar et al., 2022; Silva et al., 2022). Therefore, patients with pre-existing cardiovascular disease may experience more severe complications and adverse events after infection with neocoronavirus. In an ACE2-deficient environment, SARS-CoV-2 entry into cells induces further downregulation of ACE2, amplifying the adverse effects of the ACE/AngII/AT1 axis and attenuating the beneficial effects of the ACE2/Ang1-7 (Angiotensin1-7) /mas axis, leading to cardiomyocyte fibrosis, endothelial damage, mediating inflammatory responses and vasoconstriction, and accelerating cardiovascular diseases such as hypertension and heart failure’s further progression (Verdecchia et al., 2020; García-Escobar et al., 2021).

Hypertension, diabetes, and smoking, as risk factors for neocoronavirus infection, can increase ACE2 expression and promote viral entry into cells, leading to energy metabolism and dysfunction (Gao et al., 2021; Kato et al., 2022). This was confirmed by Kato et al., who used rat cardiomyocytes as a model for SARS-CoV-2 pseudovirus infection and found that stimulation by risk factors for cardiac disease can promote ACE2 expression and increase viral susceptibility. In addition, upregulation of ACE2 expression could be achieved by the SARS-CoV-2 S protein by promoting the formation of the TRPC3-NoX2 complex, which is an important target of viral infection and can lead to structural and functional damage of the heart, and the SARS-CoV-2 pseudovirus could promote the release of extracellular ATP through pannexin1 (Panx1), leading to increased reactive oxygen species production while promoting the formation of the TRPC3-NoX2 complex (Kato et al., 2022).

Among the isoforms of apolipoprotein E (APOE), APOE4 has been recognized as a risk factor for the cardiovascular system, which increases the risk of atherosclerosis by increasing LDL levels (Mahley, 2016; Marais, 2019). It has been found that APOE binding to ACE2 attenuates the interaction of ACE2 with viral stinger proteins, inhibits SARS-CoV-2 pseudovirus infection, and attenuates the inflammatory response (Zhang et al., 2022). However, the inhibitory effect of APOE4 was lower due to different conformational structures. In addition to this, patients carrying the APOEε4 gene have a higher susceptibility to SARS-CoV-2 and increased inflammatory factors in the serum (Zhang et al., 2022). Therefore, exploring the relationship between APOE and COVID-19 may provide new ideas to further investigate the interaction between SARS-CoV-2 and cardiovascular disease, which is expected to further accurately assess patient risk and guide treatment.

### 2.2. Cellular and molecular mechanisms

It has been more than 2 years since the prevalence of COVID-19, and with the changing trend of the disease epidemic, the focus of research has transitioned from the study of pathogenesis in the acute phase to the exploration of clinical symptoms that persist after recovery from infection. Long COVID refers to signs and symptoms that persist or develop after acute neocoronavirus infection, affect almost all organs of the body, and can lead to more than 200 different clinical manifestations (Gyöngyösi et al., 2023). Among them, cardiovascular disease is very common, often with a poor prognosis, and its cellular and molecular pathological mechanisms have been investigated by several research teams.

Neutrophils produce neutrophil extracellular traps (NETs), structures composed of granular and nuclear components that are involved in pathophysiological processes such as autoimmune and inflammatory responses, platelet activation, and the promotion of inflammatory storms and thrombotic disease (Lee et al., 2017; Cedervall, 2018). COVID-19 was found to trigger increased expression of NETs through multiple mechanisms, and NETs play an important role in the transition from acute to chronic persistent neocoronavirus infection (Zuo et al., 2020; Gyöngyösi et al., 2023). In addition, Warnatsch et al. showed that cholesterol crystals in atherosclerosis promote the release of NETs, which activate macrophages to release cytokines and lead to the further development of atherosclerotic aseptic inflammation (Warnatsch et al., 2015). Therefore, the persistence of long COVID cardiovascular symptoms is closely associated with NETs, and targeted inhibition of NETs may be an effective therapeutic option to mitigate cardiac injury. In addition to this, epigenetic reprogramming of hematopoietic progenitor cells and cellular dysregulation may be involved in the clinical manifestations of the long COVID cardiovascular system, but the above mechanisms are only highly speculative, and the exact pathological process needs to be further clarified (Gyöngyösi et al., 2023).

Numerous researchers attention has recently turned to the role played by cellular senescence in long COVID. The invasion of SARS-CoV-2 into humans is found to increase the host cell’s stress response and induce cellular senescence, which increases the pro-inflammatory, tissue-damaging senescence-associated secretory phenotype (SASP) via Toll-like receptor 3 (TLR3; Tripathi et al., 2021). SASP activates neutrophils to produce NETs, promotes platelet activation, and enhances the inflammatory response of the virus to the body. Meanwhile, SASP can expand cellular senescence through a paracrine pathway, which further leads to tissue and organ damage (Schmitt et al., 2023). Therefore, targeted inhibition of TLR3 or senescent cells is meaningful to mitigate the adverse clinical outcomes caused by COVID-19, and further exploration of this is warranted.

### 2.3. Endothelial cell dysfunction

Endothelial cells are very important for maintaining vasoconstriction and diastole, regulating the flow of cells, molecules, and fluids inside and outside the blood vessels, maintaining the balance of coagulation and fibrinolysis, and participating in immune inflammatory responses (Krüger-Genge et al., 2019). When endothelial cells become structurally and functionally impaired, it will promote the development of many cardiovascular diseases, such as atherosclerosis, coronary artery disease, and hypertension (Incalza et al., 2018). Numerous studies have shown that neocoronavirus infection can cause serious cardiovascular system complications and that patients with pre-existing cardiac disease have a higher incidence of adverse events and mortality after infection with neocoronavirus, with endothelial damage being an important part of the pathogenesis (Kang et al., 2020; Shi et al., 2020; Gu et al., 2021; Rossouw et al., 2022). Histological and pathological findings of patients who died from neointimal pneumonia suggested the presence of endothelial inflammation and degradation of endothelial cells in multiple organs throughout the body, and the presence of viral structures in endothelial cells was observed by electron microscopy (Fodor et al., 2021). Thus, the involvement of endothelial cells in the pathological development of the neocoronavirus was further confirmed.

The pathological mechanism of endothelial cell structure and dysfunction caused by SARS-CoV-2 is complex. First, the virus itself attacks and damages endothelial cells. Among the SRAS-CoV-2 proteins, S protein disrupts endothelial cell integrity, and nucleocapsid protein (NP) induces a pro-inflammatory cell phenotype that triggers the release of inflammatory factors and cytokines by binding to TLR2 in endothelial cells, triggering the NF-κB and MAPK signaling pathways (Qian et al., 2021). Both of these proteins drive viral-mediated endothelial injury. S proteins consist of S1 and S2 subunits that bind to glycosaminoglycans on vascular endothelial cells, enabling them to recognize and interact with ACE2 on the cell surface, thereby inducing viral infection of cells (Rossouw et al., 2022). Unlike cardiomyocytes, S protein-mediated viral entry into pulmonary vascular endothelial cells is followed by an upregulation of ACE2 expression and an accompanying decrease in endothelial NO synthase (eNOS) activity (Lei et al., 2021). eNOS reduction can lead to decreased NO synthesis and increased catabolism, resulting in endothelial injury (Cai and Harrison, 2000). In addition to structural damage, this experiment also demonstrated the presence of endothelial dysfunction: endothelium-dependent dilation was blocked in the pulmonary arteries of hamsters receiving a sham virus infection, whereas non-endothelium-dependent dilation was unaffected (Lei et al., 2021). An *in vitro* study showed that the endothelial permeability of brain microvascular endothelial cells in normal and diabetic mice treated with SARS-CoV-2 S protein increased in both groups, and the expression of vascular endothelial (VE)-cadherin, junctional adhesion molecule-A (JAM-A), connexin 43, and platelet endothelial cell adhesion molecule-1 (PECAM-1), which are essential for maintaining the structural integrity and normal function of the endothelium, was significantly reduced. This study demonstrated that SARS-CoV-2 can cause impaired endothelial structure and function and reduce vascular barrier function, providing strong theoretical support for the mechanism of virus-endothelial cell interaction (Raghavan et al., 2021). In addition to the above mechanisms, S proteins can activate the alternative pathway of complement (APC) to increase endothelial cytotoxicity and activate NLRP3 present in vascular endothelial cells, leading to endothelial cell dysfunction (Yu et al., 2020; Rossouw et al., 2022).

Studies have shown that in addition to direct viral damage to endothelial cells, SARS-CoV-2 can also indirectly damage endothelial cells through oxidative stress, which is a long-lasting pathological process (Fodor et al., 2021). SARS-CoV-2 can induce activation of NADPH-oxidase and promote superoxide (O_2_^−^) production, which leads to mitochondrial damage (Fodor et al., 2021). Montiel et al. found that after viral infection, damaged mitochondria can promote β-oxidation of fatty acids in vascular endothelial cells to increase oxidative stress (Montiel et al., 2022). In addition to this, oxidative stress can promote the oxidation of thiols in SARS-CoV-2 and SARS-CoV-2 proteins to disulfides, increasing viral binding to ACE2 and thus aggravating the infection (Hati and Bhattacharyya, 2020). Therefore, the reduction of oxidative stress is an essential component for the intervention and control of the recent and long-term complications of a neocoronavirus infection.

Under normal conditions, endothelial cells prevent thrombosis by inhibiting platelet aggregation and fibrin formation. Increased expression of adhesion molecules and platelet aggregation occur when severe infections lead to endothelial cell damage (Gavriilaki et al., 2020; Prasad et al., 2021; Rossouw et al., 2022). A new coronavirus infection can cause a greatly increased incidence of thrombotic events, such as pulmonary embolism and myocardial infarction, which are inextricably linked to endothelial cell dysfunction. It was found that some patients with neocoronavirus infection have different types of antibodies in their sera that activate endothelial cells to increase the expression of surface adhesion molecules such as intercellular adhesion molecule-1, E-selectin, and vascular cell adhesion molecule-1, increasing the incidence of adverse thrombotic events (Shi et al., 2022). Meanwhile, Toll receptor 7 (TLR7) on the platelet surface during SARS-CoV-2 infection binds to the single-stranded RNA of SAR2-CoV-2, accelerating endothelial damage and leading to increased thrombotic susceptibility (Rossouw et al., 2022). Severe SAR2-CoV-2 can lead to endothelial cell injury, causing excessive platelet stress, and the interaction between the two disrupts the pre-existing homeostatic balance of the vasculature, thereby causing cardiovascular system disorders by mechanisms involving microvascular occlusion, cellular oxidative stress, and the release of pro-thrombotic/pro-coagulant factors (Rossouw et al., 2022). In addition to coagulation disorders, endothelial cell dysfunction can be secondary to inflammatory responses and increased vascular permeability, leading to the development of myocardial edema and myocarditis (Gavriilaki et al., 2020; Prasad et al., 2021; Rossouw et al., 2022).

### 2.4. Insulin resistance

Insulin resistance is closely associated with cardiovascular disease and can cause myocardial fibrosis and myocardial injury and even induce cardiac insufficiency through abnormal insulin metabolism, mitochondrial dysfunction, hyperglycemia, and glucose toxicity (Jia et al., 2018; Nakamura et al., 2022). Currently, several studies have demonstrated that COVID-19 can induce the development of insulin resistance, which increases the incidence of cardiovascular disease and more severe infectious complications. Shin et al. found that SARS-CoV-2 infection can mediate an impaired insulin/insulin-like growth factor signaling pathway through interferon regulatory factor 1 (IRF1), resulting in metabolic abnormalities and tissue damage such as insulin resistance, new-onset diabetes, etc., and IRF1 expression is higher in men, diabetic, and obese populations, a group that can develop a more severe outcome of SARS-CoV-2 infection (Shin et al., 2022). He et al. demonstrated that insulin resistance is due to SARS-CoV-2 upregulation of RE1 silencing transcription factor (REST) expression to regulate the metabolic factors myeloperoxidase (MPO), apelin, and myostatin gene expression, that such metabolic disorders are not improved by the disappearance of the virus *in vivo*, and that this pathology develops over time (He et al., 2021). The above study not only provides an important reference for exploring the mechanism of interaction between COVID-19 and metabolic disorders but also provides an important theoretical basis for risk stratification and the prognosis of infected patients.

### 2.5. Iron homeostasis imbalance

Several studies have recently demonstrated the involvement of iron metabolism in the development of COVID-19. Baier et al. demonstrated for the first time that SARS-CoV-2 infection can cause an imbalance in iron homeostasis and intracellular iron accumulation, leading to decreased iron storage capacity, which induces ROS production to cause increased oxidative stress and cellular damage in cardiomyocytes (Baier et al., 2022). The interaction between SARS-CoV-2 and iron metabolism disorders was also demonstrated by Han et al. SARS-CoV-2 induces ferroptosis in human sinoatrial node pacemaker cells, causing severe lipid peroxidation, which results in pacing dysfunction and bradycardia (Han et al., 2022). Reducing intracellular iron accumulation and improving the dysregulation of iron metabolism might be important in reducing myocardial injury and arrhythmogenesis, which need further exploration and research.

### 2.6. Psychosocial factors

**Figure 1:**
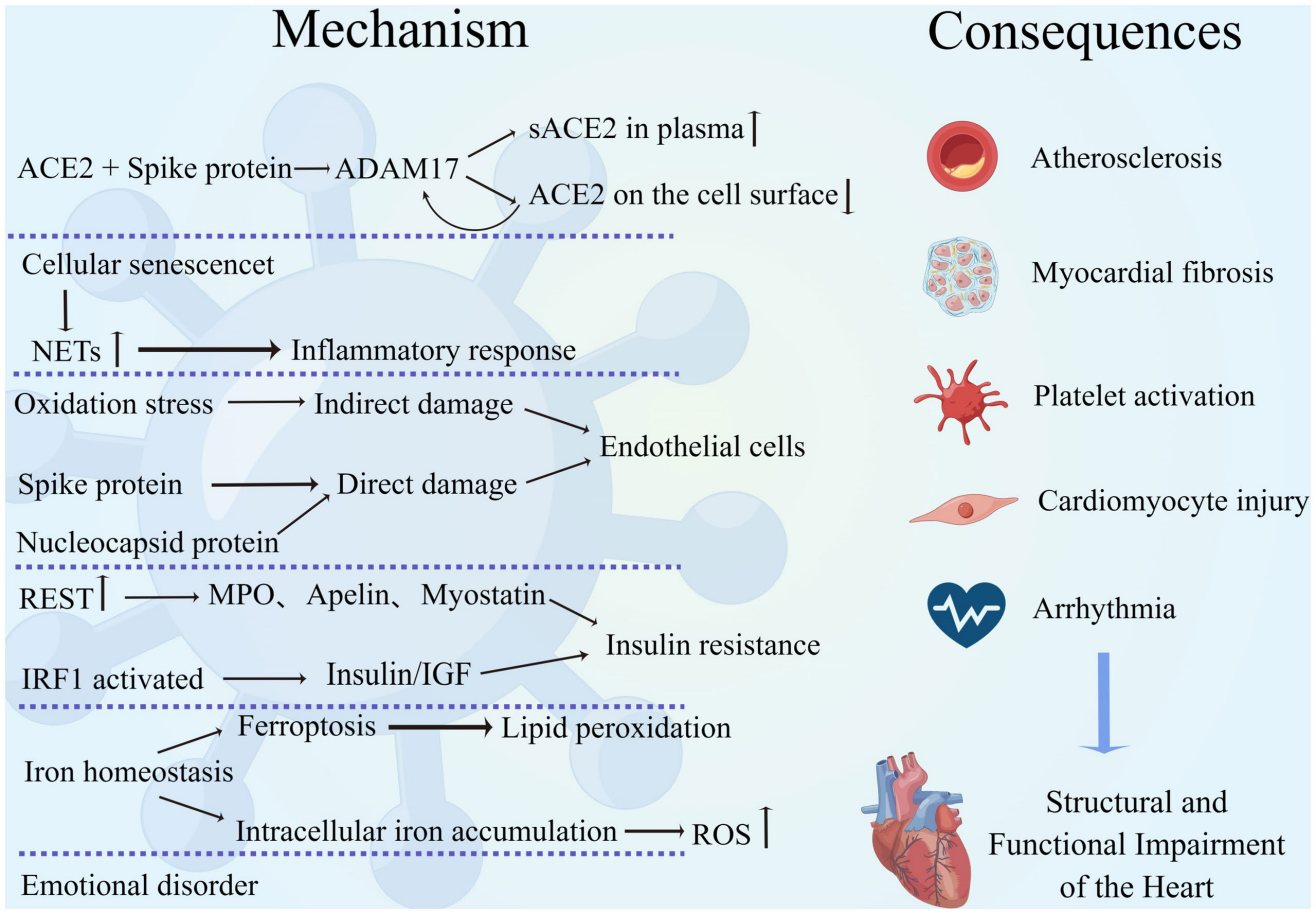
Mechanism of interaction between COVID-19 and the cardiovascular system. SARS-CoV-2 interacts with the cardiovascular system through multiple mechanisms, leading to a variety of adverse outcomes such as atherosclerosis, myocardial fibrosis, excessive platelet activation, cardiomyocyte injury, and arrhythmias, resulting in structural and functional damage to the heart. (By Figdraw).

COVID-19 has altered people’s old lifestyles, impaired physical functioning, increased economic and social stress, and put the physical and mental health of people worldwide at great risk. A study of a global meta-analysis of anxiety, depression, and stress before and during the COVID-19 pandemic showed that the prevalence of each of these negative emotions increased during the pandemic (Daniali et al., 2023). In addition to this, the COVID-19 outbreak and epidemic and some of the measures taken to reduce the further spread of the disease led to unemployment, reduced income, a lack of physical activity, and social isolation, all of which are adverse effects that are risk factors for cardiovascular disease and increase the incidence and potential for worsening of the disease (Lau and McAlister, 2021). COVID-19, affective disorders, and cardiovascular disease do not exist independently but can coexist and interact with each other, increasing the risk of adverse outcomes (Bucciarelli et al., 2022). Older adults are a vulnerable population for cardiovascular disease and COVID-19 and are susceptible to the negative effects of stressful life events. Ward et al. showed a significant increase in depression and loneliness in the elderly population during the neocoronavirus epidemic (Ward et al., 2023), while Gerhards et al. similarly demonstrated that during the COVID-19 epidemic, patients with cardiovascular disease in the elderly population were significantly more likely to be depressed and lonely compared to depression and psychological burden in healthy individuals (Gerhards et al., 2023). Interestingly, it has been documented that the correlation between life isolation and cardiovascular disease was stronger in participants < 65 years of age compared to those >65 years of age (Holt-Lunstad et al., 2015; Gronewold et al., 2020). In general, the potential psychological disorders brought on by neocoronavirus infection cannot be ignored and can increase the risk of cardiovascular disease through a variety of physiological mechanisms in the body, despite the fact that there are varied results regarding the risk of cardiovascular disease at various ages under the influence of significant social events (Gronewold et al., 2021; Figure 1).

## 3. Differences and analysis of cardiovascular system diseases combined with COVID-19 in different populations

### 3.1. Gender

Gender differences are one of the key determinants of disease progression and outcome and have received increasing attention and research. Several studies have found that among patients infected with SARS-CoV-2, disease morbidity, severity, and mortality appear to be higher in men than in women (Chen et al., 2020; Marik et al., 2021; Wehbe et al., 2021; Torres et al., 2023). Cardiovascular system disease is one of the common comorbidities and complications of neocoronavirus infection, and their interaction can increase the risk of death in patients (Wehbe et al., 2020; Woodruff et al., 2023). Recently, a series of explorations have been conducted regarding gender differences in cardiac disease combined with COVID-19. The prevalence of COVID-19 in combination with cardiovascular disease is higher in male patients worldwide (Gebhard et al., 2020). The increased mortality associated with acute myocardial infarction in COVID-19 patients is higher in men than in women (Yeo et al., 2023). Isath et al. found more men among patients with COVID-19 combined with heart failure by analyzing the baseline characteristics of patients admitted with heart failure in the United States (Isath et al., 2023b). The above studies suggest that men may have greater susceptibility to and more severe adverse clinical outcomes from neocoronavirus infections.

The reasons for this are the involvement of ACE2 and type II transmembrane serine protease (TMPRSS2) expression, sex hormones, and immune and inflammatory responses. ACE2 and TMPRSS2 are key factors in promoting SARS-CoV-2 entry into cells (Hoffmann et al., 2020), and it was found that plasma concentrations of ACE2 were higher in males, while TMPRSS2 is more expressed in males and regulated by androgens (Okwan-Duodu et al., 2021), which may lead to an increased initial viral load (Viveiros et al., 2022). When SARS-CoV-2 binds to ACE2, it activates metalloproteinase 17 (ADAM17), which induces ACE2 membrane shedding, exacerbates the accumulation of AngII, and diminishes the cardioprotective effects of ACE2 (Gheblawi et al., 2020). TLR7 recognizes single-stranded RNA and promotes interferon production, playing an important role in the immune response to new coronary infections. Females can express higher amounts of TLR7, thereby increasing resistance to viruses (Bienvenu et al., 2020; Wehbe et al., 2021). Sex hormone-dependent innate immunity leads to gender differences in the face of viral infections. Estradiol enhances the antiviral response by increasing the number of neutrophils and natural killer cells and decreasing pro-inflammatory cytokines, whereas androgens have immunosuppressive effects (Viveiros et al., 2021; Bechmann et al., 2022; Brandi, 2022). Recently, the first exploration regarding the effect of COVID-19 on patients with stress cardiomyopathy was conducted by Hajra et al. This study found that stress cardiomyopathy combined with COVID-19 was predominantly in men, and, interestingly, stress cardiomyopathy was more common in women (Hajra et al., 2023). It is worth noting that there are still flaws and controversies regarding the above mechanisms and studies. For example, it is too limited to consider only the ACE2 expression and ignore ACE2’s own function. Differences in sex hormone levels in older patients may affect the observed sex differences. Both confounding factors and vaccination may affect the outcome of the studied patients.

Currently, the exploration of gender differences for COVID-19 combined with CVD is more based on physiological mechanisms. However, the impact of the new coronavirus on the psychological aspects of humans cannot be ignored. It has been found that the COVID-19 pandemic can lead to more stress in women from various aspects such as social, economic, work, and family, exacerbating the development of psychological disorders such as depression and anxiety and thus leading to an increased risk of CVD (Gulati and Kelly, 2020; Bucciarelli et al., 2022).

Gender differences in COVID-19 combined with CVD should be investigated further, not only in terms of physiological mechanisms but also to emphasize the importance of psychosocial factors in disease development. Not only will this allow for better risk stratification for patients, but it will also allow for more refined and accurate treatment.

### 3.2. Age

Age is an important risk factor for increased mortality in patients with COVID-19, and older adults usually have a higher susceptibility as well as more severe clinical outcomes (Bonanad et al., 2020; Zhou et al., 2020; Torres et al., 2023). As previously mentioned, COVID-19 can increase the incidence of cardiovascular disease through endothelial damage, an immune inflammatory response, and coagulation abnormalities, and patients with underlying cardiac disease or the presence of cardiovascular disease risk factors are more likely to experience serious outcomes (Tian et al., 2020). Several studies have demonstrated that the risk of cardiovascular complications is greatly increased in the elderly population with a COVID-19 infection. Pellicori et al. found that some patients with COVID-19 can develop different types of cardiovascular complications during hospitalization and that the risk of CVD increases with age (Pellicori et al., 2021). Hajra et al. demonstrated that advanced age is a risk factor for stress cardiomyopathy and an independent predictor of mortality in patients with combined COVID-19 (Hajra et al., 2023). Hypertension is a common cardiovascular comorbidity in patients with COVID-19 (Harrison et al., 2021; Pellicori et al., 2021), and its pathogenesis is closely related to ACE2. ACE2 is both an important target of SARS-CoV-2 infected cells, and its elevated expression may be associated with an increased risk of infection while acting as a protective factor of the cardiovascular system to mitigate the deleterious effects of AngII. SARS-CoV-2-infected cells result in a further reduction of ACE2, while downregulation of ACE2 expression leads to more severe cardiac dysfunction. Thus, elderly people with down-regulated ACE2 expression have a more severe infection outcome (AlGhatrif et al., 2020). Older adults with cardiometabolic disease (CMD) have more severe adverse outcomes when infected with neocoronavirus, making social isolation particularly important for this high-risk population. However, DOVE et al. found that this population is more vulnerable to the negative psychological effects of social isolation, which can exacerbate the severity of cardiovascular disease, which requires us to weigh the pros and cons of each aspect in our clinical work and give targeted treatment and prevention recommendations (Dove et al., 2022).

Age is an uncontrollable risk factor for COVID-19 and cardiovascular disease, and exploring the link between age and disease pathogenesis will facilitate better risk stratification and management of patients. The physical and mental health of the elderly is highly vulnerable to disease itself and social factors; therefore, both “body” and “mind” should be the focus of our care and support for this population.

### 3.3. Metabolic syndrome

Metabolic syndrome (MetS) is a complex group of metabolic disorder syndromes, including obesity, dyslipidemia, hyperglycemia, and hypertension, which are closely associated with the development of cardiovascular diseases (Hamjane et al., 2020; Fahed et al., 2022). Epidemiology has shown that the prevalence of MetS has increased in all regions of the world, posing a huge health, economic, and medical burden and becoming an important health problem in modern society (Saklayen, 2018; Li et al., 2022). The pathogenesis of MetS is closely related to insulin resistance, chronic inflammation, mitochondrial dysfunction, and neurological activation, and interestingly, the above pathological processes are also involved in the development of cardiovascular disease and COVID-19 (Fahed et al., 2022; Dissanayake, 2023). A large body of evidence suggests that MetS leads to an increased risk of death and the development of serious complications in patients with COVID-19, often with a worse clinical outcome when combined with cardiovascular disease (Rico-Martín et al., 2021; Frere and tenOever, 2022).

**Figure 2:**
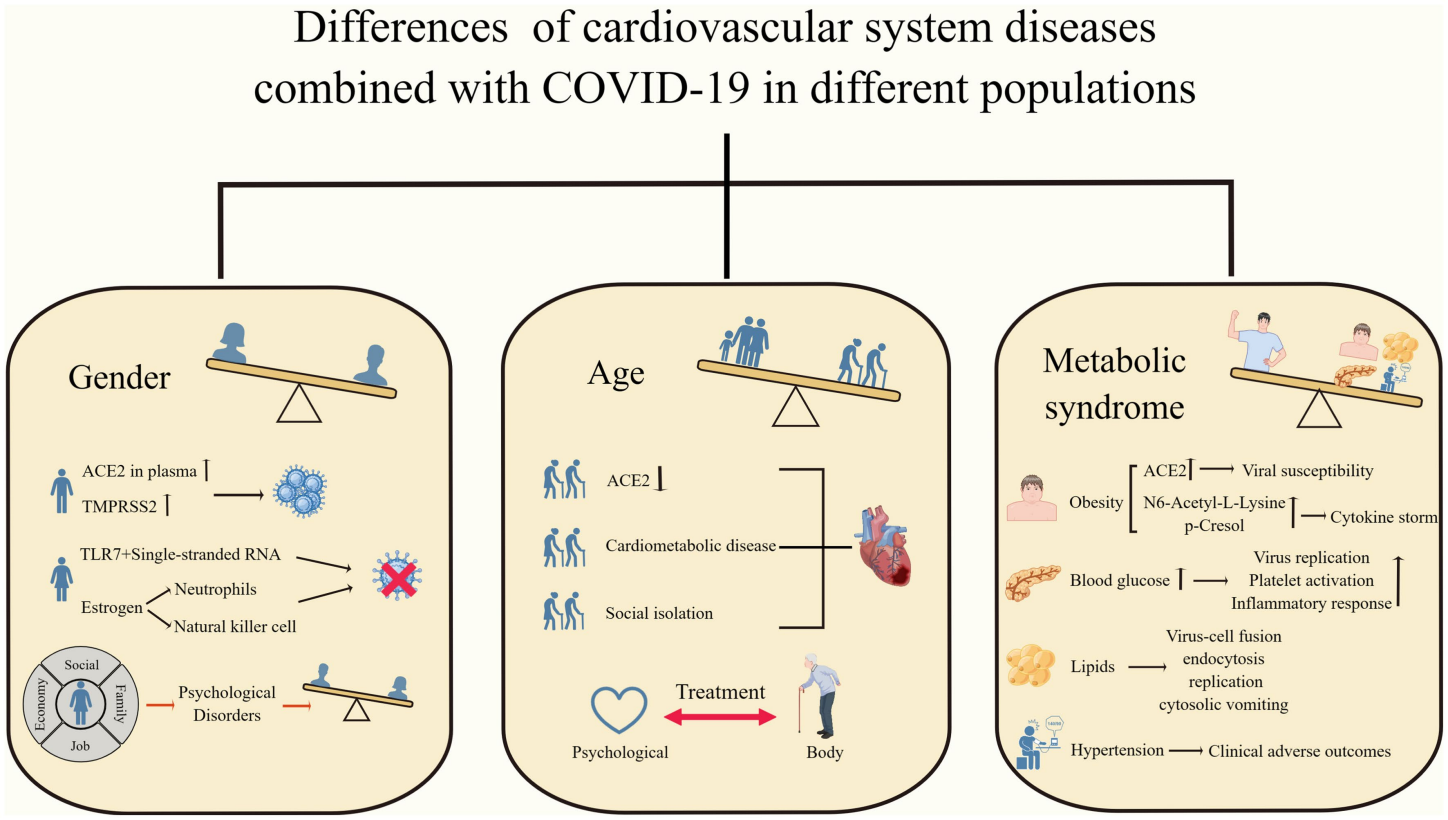
Differences in cardiovascular system diseases combined with COVID-19 in different populations. Men, older adults, and patients with metabolic syndrome have worse clinical outcomes when faced with COVID-19 combined with cardiovascular disease. (By Figdraw).

Obesity, as a manifestation of the metabolic syndrome, can enhance SARS-CoV-2 susceptibility by increasing ACE2 expression. In addition, it was found that obese patients have elevated levels of n6-acetyl-l-lysine and p-cresol, which induce cytokine storm production and exacerbate the severity of COVID-19 (Jalaleddine et al., 2022). Protein lysine acetylation leads to impaired cardiac energy metabolism, and p-cresol enhances the risk of atherosclerosis and thrombosis in uremic patients (Jalaleddine et al., 2022). Increased blood glucose increases the risk of SARS-CoV-2 infection and the possibility of cardiovascular system disease through mechanisms such as the promotion of viral replication, platelet activation, and an excessive inflammatory response. Conversely, COVID-19 infection can cause disturbances in glucose regulation and induce serious complications (Aluganti Narasimhulu and Singla, 2022). Lipids are not only the structural basis of cells but also involved in several physiological or pathological activities in the body, which are essential for the development of the cardiovascular system and COVID-19. Lipid metabolism plays an important role in the processes of virus-cell fusion, endocytosis, replication, and cytokinesis, so statins are beneficial in reducing viral infections, while their plaque stabilizing effect reduces the probability of cardiovascular disease (Abu-Farha et al., 2020). Much controversy remains regarding the interaction between hypertension and COVID-19. Studies suggest that hypertension can occur in the acute phase of SARS-CoV-2 infection or as a sequela and has the potential to be a long-term predictor of COVID-19, but the evidence against the above view is not sufficient, and there are no studies to prove that pre-existing hypertension is an independent risk factor for increased mortality in COVID-19 (D'Elia et al., 2023; Matsumoto et al., 2023; Shibata et al., 2023). However, it is undeniable that hypertension, as a systemic disease, can lead to serious adverse clinical outcomes in patients with COVID-19, posing a significant threat to individual health and the global health care system (Abdalla et al., 2023; Figure 2).

## 4. Summary and outlook

The incidence of cardiovascular disease was significantly higher in the SARS-CoV-2 infection state, with significant differences across populations. COVID-19 patients with preexisting cardiovascular disease risk factors or cardiac disease had worse clinical outcomes. In addition to ACE2-mediated viral entry, endothelial dysfunction, iron homeostasis imbalance, and psychosocial factors involved in pathological development, severe electrolyte disturbances, respiratory failure, and impaired mitochondria can also lead to cardiac involvement (Wu et al., 2020; Adeghate et al., 2021; Chang et al., 2023). Take consideration of the spreading time of COVID-19 and the multiple variant strains, the long-term effects of SARS-CoV-2 on the heart are not yet known, and we still need to conduct long-term follow-up and a lot of research and exploration.

## Author contributions

YZ and XH reviewed the literature and drafted this review. CL, YL, JC, and YW reviewed the literature, gave critical comments, and revised the manuscript. BA gave critical comments and revised the manuscript. All authors contributed to the article and approved the submitted version.

## Funding

The study was supported by the National Natural Science Foundation of China (grant numbers: 82170362 to YW and 82000347 to CL). This study was also supported by Jilin Province Science and technology development plan project (grant numbers: 20230508061RC to YW and YDZJ202301ZYTS441 to CL), China Postdoctoral Science Foundation (2021M691209 to YW), and Jilin Medical and Health Talents Special (JLSWSRCZX2021-061 to YW).

## Conflict of interest

The authors declare that the research was conducted in the absence of any commercial or financial relationships that could be construed as a potential conflict of interest.

## Publisher’s note

All claims expressed in this article are solely those of the authors and do not necessarily represent those of their affiliated organizations, or those of the publisher, the editors and the reviewers. Any product that may be evaluated in this article, or claim that may be made by its manufacturer, is not guaranteed or endorsed by the publisher.
